# Influence of Dietary Habits and Mediterranean Diet Adherence on Sleep Quality during Pregnancy. The GESTAFIT Project

**DOI:** 10.3390/nu12113569

**Published:** 2020-11-20

**Authors:** Marta Flor-Alemany, Teresa Nestares, Inmaculada Alemany-Arrebola, Nuria Marín-Jiménez, Milkana Borges-Cosic, Virginia A. Aparicio

**Affiliations:** 1 Department of Physiology, University of Granada, 18071 Granada, Spain; floralemany@ugr.es (M.F.-A.); virginiaparicio@ugr.es (V.A.A.); 2 Institute of Nutrition and Food Technology (INYTA), Biomedical Research Centre (CIBM), University of Granada, 18016 Granada, Spain; 3 Sport and Health University Research Institute (IMUDS), 18007 Granada, Spain; nuriaproyecto@gmail.com (N.M.-J.); milkanaa@hotmail.com (M.B.-C.); 4 Department of Developmental and Educational Psychology, Faculty of Education and Sports Sciences, University of Granada, 52005 Melilla, Spain; alemany@ugr.es; 5 Department of Physical Education and Sport, Faculty of Sport Sciences, University of Granada, 18071 Granada, Spain

**Keywords:** dietary pattern, gestation, diet, sleep quality, Pittsburgh sleep quality index

## Abstract

We examined the association of the dietary habits and the Mediterranean diet (MD) adherence with sleep quality during pregnancy. A food frequency questionnaire and the Mediterranean Food Pattern were employed to assess dietary habits and MD adherence, respectively. Sleep quality was assessed with the Pittsburgh Sleep Quality Index (PSQI) global score (*n* = 150; mean age 32.9 ± 4.6 years). A higher consumption of fruits was associated with better sleep quality at the 16th gestational week (g.w.; *p* < 0.05). A greater olive oil consumption and a higher MD adherence were associated with better sleep quality at the 16th and 34th g.w. (all, *p* < 0.05). Contrarily, a higher red meat and subproducts consumption was associated with worse sleep quality at the 34th g.w. (*p* < 0.05). The group with the highest adherence to the MD (Tertile 3) showed better sleep quality than the group with the lowest adherence (Tertile 1) at the 16th and 34th g.w. (both, *p* < 0.05). A higher adherence to the MD, a greater intake of fruits and olive oil and a lower intake of red meat and subproducts were associated with better sleep quality along the pregnancy course, especially among sedentary women.

## 1. Introduction

Sleep disturbances are common complaints during pregnancy, with recent studies suggesting that almost 50% of expectant mothers experience poor sleep quality, with rates close to 75% by the third trimester of pregnancy [1,2,3]. Assessments of sleep quality during pregnancy might be clinically relevant given the evidence that poor sleep quality is linked with an array of adverse health outcomes including inflammation, metabolic syndrome and type 2 diabetes [1,4,5,6]. Moreover, recent hypotheses suggest that poor sleep quality is associated with negative birth outcomes such as increased odds of preterm birth, caesarean section, shorter length of gestation and longer labor [1,6,7], whereas good sleep quality is associated with a better Apgar score among neonates and birth weight [8].

Considering the impact of sleep-related habits on adverse health outcomes, it is crucial to investigate and identify potential dietary determinants of sleep quality during pregnancy [9]. Among the many factors studied that could exert an influence on sleep quality, diet seems to have an impact on both sleep quality and its related health outcomes [4]. Indeed, sleep and diet are strongly interrelated, with recent studies [4,10,11,12] suggesting a bi-directional association: poor sleep quality may negatively affect dietary habits by reducing overall diet quality and increasing appetite and caloric intake [11], while at the same time food choices might influence sleep quality [12]. With this in mind, poorer dietary patterns, such as those characterized by a high fat and sugar content, have been linked to worse sleep quality in all age groups [13,14,15]. On the contrary, cross-sectional studies [9,11,16] have shown that diets with a high intake of fruits and vegetables and a lower intake of saturated fatty acids, such as the Mediterranean diet (MD), might be beneficial for sleep quality in the adult population. Although these observations helped to establish a sleep–diet relation, little is known about how the MD adherence and its components may be linked to measures of sleep quality in pregnant women. Therefore, the aim of the present study was to explore the association of dietary habits and the MD adherence with sleep quality during pregnancy.

## 2. Materials and Methods

### 2.1. Study Design and Participants

**Figure 1:**
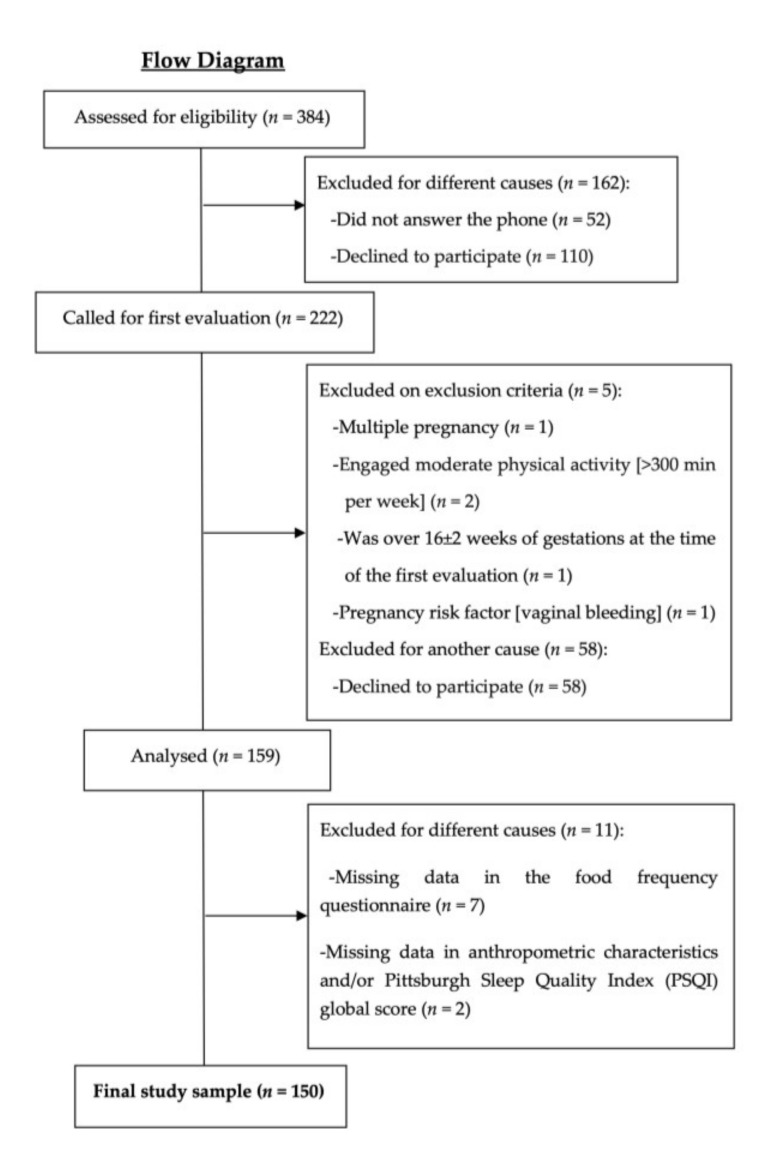
Flow diagram of the study participants.

The present cross-sectional study forms part of the GESTAFIT project, where a novel exercise intervention was conducted [17]. The entire methodology of the project, the inclusion–exclusion criteria and the sample size calculation to detect clinically meaningful changes in the intervention program have been published elsewhere [17]. The required sample size was only determined for the primary outcome (maternal weight gains and maternal/neonatal glycemic profile) of the GESTAFIT project. A total of 159 Spanish pregnant women (32.9 ± 4.6 years old) enrolled in this study in three waves (from November 2015 to March 2017), for feasibility reasons. The participants were recruited between the 11th to 13th gestational weeks (g.w.) at the “San Cecilio” University Hospital (Granada, Spain) during their first gynecologist checkup. This study was approved by the Ethics Committee on Clinical Research of Granada, Regional Government of Andalusia, Spain (code: GESFIT-0448-N-15). The procedures described in the manuscript have been carried out in accordance with the Code of Ethics of the World Medical Association (Declaration of Helsinki). From the 159 pregnant women recruited, this cross-sectional study included 150 women (mean age 32.9 ± 4.6 years) at the 16th g.w. who had valid data in the food frequency questionnaire and the Pittsburgh Sleep Quality Index (PSQI; Figure 1). From the 150 pregnant women, 32 had missing data in the food frequency questionnaire and/or the PSQI global score at the 34th g.w. As a result, a total of 118 pregnant women were included for the present analyses at the 34th g.w.

### 2.2. Sociodemographic Characteristics

The evaluation procedures were carried out at the 16th and 34th g.w. at the Sport and Health University Research Institute (iMUDS). The assessments were conducted in a single day. At the 16th g.w., data regarding sociodemographic and lifestyle characteristics (i.e., age; educational, marital and working status; number of children; smoking habit and physical or psychological disease diagnosis) were collected through an initial survey (anamnesis).

### 2.3. Maternal Anthropometry and Body Composition

Pre-pregnancy body weight was self-reported. Body weight and height were measured using a scale (InBody R20; Biospace, Seoul, Korea) and a stadiometer (Seca 22, Hamburg, Germany), respectively. Those measurements were employed to calculate pre-gestational body mass index and body mass index at the 16th gestational week as weight (kg) divided by squared height (m^2^).

### 2.4. Dietary Assessment

Dietary habits were collected by using the food frequency questionnaire designed by Mataix et al. [18]. The same trained nutritionist administered the questionnaires to pregnant women at the 16th and 34th g.w.

The Mediterranean Food Pattern (MFP; a Mediterranean adherence score) was constructed with the data obtained from the food frequency questionnaire [18]. We employed this dietary index because it was previously associated with lower cardiometabolic risk along the pregnancy course in this study sample (submitted data). The MFP was calculated based on previously published literature [19]. It consists of eight elements (olive oil, fiber, fruits, vegetables, fish, cereals, meat and alcohol) ranging from 5 to 40. Notwithstanding, alcohol consumption was not considered when calculating the total score. Thus, the maximum score ranges from 4 to 35, where higher scores indicate greater MD adherence.

### 2.5. Sleep Quality

The Spanish version of the PSQI [20] was employed to assess sleep quality, since the PSQI has been shown to have a good construct validity among pregnant women [21]. The PSQI is a self-rated questionnaire that measures sleep quality from the previous month, comprising 19 questions divided into seven categories: subjective sleep quality, sleep latency, sleep duration, sleep disturbances, sleep efficiency, use of sleep medication and daytime dysfunction. Each component scores from 0 to 3, with a total score that ranges from 0 to 21 with lower values indicating better sleep quality [20]. The suggested cutoff is 5 points differentiating “good” from “bad” sleepers [20].

### 2.6. Statistical Analyses 

Descriptive statistics (mean (standard deviation) for quantitative variables, and number of women (%) for categorical variables) were employed to describe participants’ sociodemographic characteristics. The distribution of the data was examined for all the study variables, and the PSQI global score showed a skewed distribution that could not be normalized after several transformations (e.g., logarithmic transformations). Subsequently, we performed Spearman’s correlation analysis between the dietary habits, the MFP score and the PSQI global score at the 16th and 34th g.w. Differences between dietary habits, MFP and PSQI global score by the g.w. (16th g.w. versus 34th g.w.) were tested using the Wilcoxon nonparametric test. The PSQI global score was compared across tertiles using the Kruskal–Wallis test. Post-hoc multiple comparisons with Bonferroni’s correction were applied to examine pairwise differences between groups (e.g., Tertile 1 vs. Tertile 3). In order to avoid the discrepancies noted in the literature among the large range of cutoff points for the different tools employed to assess dietary patterns during pregnancy, the MFP was also dichotomized using the 50th percentile with participants being categorized as having low or high adherence, as performed in previous studies [22,23] (Figure S1). Subsequently, the PSQI global score was compared between these dietary indices groups by using the Mann–Whitney U nonparametric test. We performed additional analyses to further explore whether several factors including pre-gestational BMI and the concurrent physical exercise program, which was carried out in the GESTAFIT project [17], exerted an influence on the studied associations. Spearman’s correlations were employed to assess the association between the dietary habits, the MFP and the PSQI global score at the 16th and 34th g.w. according to pre-gestational BMI categories and exercise intervention (intervention or control). All analyses were performed using the Statistical Package for Social Sciences (IBM SPSS Statistics for Windows, version 22.0, Armonk, NY, USA); the level of significance was set at *p* < 0.05.

## 3. Results

Sociodemographic characteristics of the participants are shown in Table 1.

Spearman’s correlation analysis assessing the association of dietary habits and the MD adherence with the PSQI global score at the 16th and 34th g.w. is shown in Table 2. At the 16th g.w., a higher consumption of fruits, olive oil and a higher MD adherence were associated with a lower PSQI global score (i.e., better sleep quality; *p* = 0.008, *p* = 0.048 and *p* = 0.039, respectively). In addition, a higher red meat and subproducts consumption was associated with a higher PSQI global score with borderline significance (i.e., worse sleep quality; *p* = 0.078). At the 34th g.w., a higher consumption of olive oil and a higher MD adherence were associated with a lower PSQI global score (*p* = 0.038 and *p* = 0.001, respectively). A higher red meat and subproducts consumption was associated with a greater PSQI global score (*p* = 0.032).

**Figure 2:**
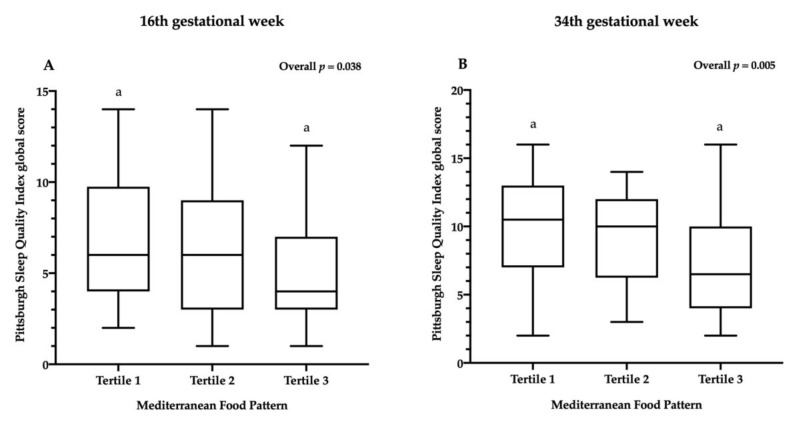
Pittsburgh Sleep Quality Index global score by tertiles of Mediterranean Food Pattern adherence. Box plots demonstrating median, upper and lower quartiles and the minimum and maximum Pittsburgh Sleep Quality Index global scores at the 16th (*n* = 150) and 34th (*n* = 118) gestational weeks. a—indicates a significant difference (*p* < 0.05) between groups. Pairwise comparisons were performed with Bonferroni‘s adjustment. (**A**) Pittsburgh Sleep Quality Index global score by the Mediterranean Food Pattern tertiles at the 16th gestational week. (**B**) Pittsburgh Sleep Quality Index global score by the Mediterranean Food Pattern tertiles at the 34th gestational week.

The PSQI global score at the 16th and 34th g.w. by tertiles of the MFP is shown in Figure 2. Pairwise comparisons showed that the group with the highest score (Tertile 3) in the MFP had a lower PSQI global score than the group with the lowest score (Tertile 1) at the 16th g.w. and 34th g.w. (*p* = 0.038 and *p* = 0.005, respectively).

The PSQI global score at the 16th and 34th g.w. according to the 50th percentile of the MFP [19] is shown in Figure S1. The group with the highest score (above the 50th percentile) in the MFP [19] had a lower PSQI global score than the group with the lowest score (below the 50th percentile) at the 16th and 34th g.w. (*p* = 0.008 and *p* = 0.005, respectively).

Differences between the dietary habits, the MFP and the PSQI global score by g.w. (16th g.w. versus 34th g.w.) are shown in Table S1. Regarding dietary habits, pregnant women at the 34th g.w. had higher intake of fruits, vegetables and whole dairy products (*p* = 0.010, *p* = 0.014 and *p* = 0.044, respectively). No differences were found regarding MFP adherence (*p* > 0.05). In addition, pregnant women at the 34th g.w. had a higher PSQI global score (*p* < 0.001).

The association between the dietary habits, the MFP and the PSQI global score at the 16th and 34th g.w. according to pre-pregnancy BMI categories and the exercise intervention (intervention or control) are shown in Tables S2 and S3. In the control group, a higher consumption of fruits was associated with better sleep quality at the 16th g.w. (*p* < 0.01). Olive oil and a higher MFP were associated with better sleep quality at the 16th and 34th g.w. (all, *p* < 0.05). All the previous associations were not significant in the intervention group (*p* > 0.05). Regarding pre-pregnancy BMI categories, a higher intake of fruits and olive oil were associated with better sleep quality at the 16th g.w. in normal-weight pregnant women and overweight/obese participants, respectively (both, *p* < 0.05). At the 34th g.w. a higher intake of olive oil and MD adherence were associated with better sleep quality among normal-weight participants (both, *p* < 0.05). A higher MD adherence was associated with better sleep quality in overweight/obese participants at the 34th g.w. (*p* < 0.05).

Differences between the PSQI global score by exercise intervention (control versus intervention) are shown in Table S4. No differences between groups were found regarding the PSQI global score by exercise intervention (*p* > 0.05).

## 4. Discussion

The main finding of the present study is that a greater adherence to the MD was associated with better sleep quality during both the 16th and 34th g.w., especially among sedentary pregnant women. In addition, a greater consumption of fruits and olive oil and a lower intake of red meat and subproducts (i.e., beef, pork, viscera and cold meat products) were associated with better sleep quality along gestation. Moreover, pregnant women with the highest adherence to the Mediterranean dietary pattern (Tertile 3) showed better sleep quality than the groups with the lowest scores (Tertile 1) during both the 16th and 34th g.w.

Sleep quality is often compromised in pregnant women and aggravated over the course of pregnancy [24]. A recent study [25] reported that 47% of pregnant women had poor sleep quality (as measured by the PSQI) between the 12th and 20th g.w., similar to our results for the 16th g.w. (48%). Moreover, sleep quality significantly decreased from second to third trimester, with 75% of pregnant women reporting poor sleep quality at the 34th g.w., which is in agreement with a previous study that showed that 75–83% of pregnant women had poor sleep quality in the third trimester of pregnancy (7–8 months) [3].

Comparing the early second trimester with the third trimester, we observed a significantly higher intake of fruits, vegetables and dairy products in the third trimester, as previously reported [26]. It is possible that participants might have increased their fruit, vegetable and dairy product intakes due to nutritional advice, which usually promotes fruit, vegetable and dairy consumption in order to meet the nutritional requirements of pregnancy [27]. However, our results showed that adherence to the Mediterranean diet remained unchanged across pregnancy. This finding suggests that food behavior of our sample did not change during gestation, which concurs with previous studies [28,29]. Moreover, during early gestation, food intake can be often affected by nausea and vomiting, physiological phenomena linked to hormonal changes during this period [30]. However, women recruited in this study were all past the 13th week of gestation, which could partially explain the lack of differences between food habits between different gestational stages.

It has been established that poor sleep quality negatively affects dietary habits by reducing overall diet quality and increasing appetite and caloric intake [11]. Notwithstanding, recent data also suggest a bi-directional association by which food choices might positively influence sleep quality [12]. Recent studies [9,11] showed an association between the adherence to the Mediterranean dietary pattern and sleep quality, suggesting that plant-rich diets might be beneficial for sleep in the adult population. However, evidence in pregnant women is scarce. A study conducted by Chang et al. [31] on overweight and obese pregnant women showed direct associations between sleep disturbances and dietary fat intake and also between shorter time taken to fall asleep and a higher fruit and vegetable intake. Nonetheless, neither diet quality nor dietary patterns were included in these studies. In agreement with our findings, a more recent study [24] showed that better sleep quality was associated with greater diet quality and a greater adherence to a dietary pattern based on fruits, vegetables and rice. In the present study sample, a higher MD (a diet high in fruits, vegetables and fiber and low in saturated fatty acids) adherence was associated with better sleep quality over the course of pregnancy.

Further, participants with the highest MD adherence (Tertile 3) had better sleep quality during the pregnancy course than the groups with the lowest scores (Tertile 1). This concurs with a previous study in a non-pregnant adult population in which individuals with a greater adherence to the Mediterranean dietary pattern presented overall better sleep quality compared to those with less adherence [9]. Moreover, if such an eating pattern influences sleep during pregnancy, it is not clear which specific component or components of the Mediterranean dietary pattern would exert a stronger influence. To further explore this issue, we also studied the different food groups that comprise the Mediterranean dietary pattern, finding that a higher intake of fruits and olive oil and a lower intake of red meat and subproducts were associated with better sleep quality during pregnancy. A previous study [9] checked if any of the MD components alone could explain the association of the MD score with better sleep quality, suggesting that olive oil consumption itself might play an independent role in sleep quality, which is highly in agreement with our results. Regarding fruits, it has been suggested that the odds of meeting or exceeding the sleep recommendations (i.e., 7–9 h per day for adults aged 18–64 years old) increase by 12% in pregnant women for every additional fruit serving consumed [32]. Similarly, a study conducted in women within 5 years of childbirth found that women with longer sleep duration (≥ 9 h) had poorer overall diet quality, a lower intake of fruits and a higher intake of calories from solid fats and added sugar, compared to women with an adequate sleep duration (7–8 h) [33]. Evidence also suggests that diets rich in fats and carbohydrates, with a tendency to include snacks between meals, are associated with poorer sleep quality and fewer sleeping hours in the general population [13,14,34]. The MFP, which was employed to calculate the MD adherence, does not directly assess sugary food intake. For this reason, the sweets variable (including soft drinks, preserved juices, biscuits, baked goods and chocolate) was additionally calculated, as it represents an important component of unhealthy dietary habits. Sweets intake in this study sample was slightly greater than one serving per day, an amount that is within the recommended intake of sugary foods (< 3 servings/day) accordingly to the final nutritional objectives for Spanish population [35]. This result could be due to the limitation of the items of the food frequency questionnaire itself or to underreporting but could also be derived from the wish of the mothers to follow healthier dietary patterns during pregnancy, avoiding highly processed foods. Moreover, the observed lower sugary food intake in this group could also explain why no correlations were found between them and the PSQI global score.

In this study sample, a higher intake of red meat and subproducts were associated with poorer sleep quality along gestation. Similarly, a study conducted by Lana et al. [36] suggested that a high protein intake derived from meat (white or red meat) was associated with poor sleep quality in the non-pregnant adult population. The detrimental effect of red meat and subproducts on sleep quality might be exerted through the protein content of meat as previously stated [36]. The effects of protein on sleep quality could be related to two amino acids (tryptophan and tyrosine) and their capacity to synthesize melatonin, serotonin and dopamine (involved in the sleep–wake cycle) [36]. It has been suggested that a high consumption of protein could reduce the blood circulation ratio of tryptophan/tyrosine, which could result in a lower synthesis of brain sleep inductors and consequently a deterioration of sleep parameters, which is in agreement with our findings [36,37,38]. Other food groups that are naturally rich in protein and were tested in this study (e.g., dairy, poultry) did not show significant results between them and the PSQI global score. Due to the fact that red meat and subproducts intake was the most consumed group of meat, this finding might overlap the potential influence of other sources of animal protein.

Previous literature showed that BMI and exercise were associated with sleep quality [39,40,41]. We further studied how these variables affected the association between MD adherence and sleep quality. We found that a higher intake of fruits and olive oil (at the 16th g.w.) and a higher consumption of olive oil and a higher MD adherence (at the 34th g.w.) were associated with better sleep quality independently of pre-pregnancy BMI categories. Regarding the exercise intervention, we found that in the control group a higher consumption of fruits, olive oil and a higher MD adherence were associated with better sleep quality along the pregnancy course. Nevertheless, all of the previous associations disappeared in the intervention group, suggesting that the diet was more effective in improving sleep quality in the control sedentary group. This might be partially explained by the effect that the exercise training could have had by itself regardless of maternal diet. Previous studies [42,43] have reported that after a period of 8–10 weeks of concurrent training, sleep quality improved, suggesting that the improvements could lead to a state of the melatonin hormone being secreted by the allergic pineal glands, which has a hypnotizing effect with central body temperature. Anabolic activity is also stronger during sleep, whereas the catabolic activity is more intense during vigilance. Therefore, for a possible balance of energy, the body consumes more energy to relax, and the body tends to increase sleep duration.

It has been suggested that the high isoflavone and tryptophan content of plant-based diets (e.g., Mediterranean diets) may be the mechanism by which plant foods enhance sleep quality [11]. Interestingly, in a subsample of participants from the PREDIMED study, participants in the two Mediterranean diet groups showed an increment in tryptophan concentrations, and this was related to lower non-stroke outcomes [44]. The authors suggested that changes in tryptophan may be involved in the cardioprotective effects of the Mediterranean diet [44]. Sleep and sleep-related metabolite derivatives of tryptophan, melatonin and serotonin were not measured in this study. Nevertheless, given our understanding of tryptophan metabolism, sleep improvements may have further played a role in this result [11].

When considering the results of the present study, some limitations ought to be kept in mind. Firstly, the cross-sectional design of the study provides information without a clear cause–effect identification. As a result, we cannot determine whether a healthier diet affects sleep quality or, on the contrary, sleep features lead to unhealthy dietary behaviors. Secondly, since we employed a food frequency questionnaire in order to assess dietary habits, we are aware of its recall bias and its lower accuracy when compared to a 24 h food diary. Nonetheless, the food frequency questionnaire (which is widely employed in nutritional epidemiology) was conducted by the same trained nutritionist along the pregnancy course. Importantly, both sleep quality and the dietary adherence were self-reported. While PSQI is a widely employed tool validated in pregnant population [21], it is not as valid as an objective measure of sleep such as polysomnography.

## 5. Conclusions

The present study provides some evidence linking the Mediterranean dietary pattern to better sleep quality during pregnancy, especially among sedentary women. Specifically, a higher intake of fruits and olive oil, a lower intake of red meat and subproducts and a greater adherence to the Mediterranean dietary pattern are associated with better sleep quality along the pregnancy course. Given the limited number of studies available, further research is warranted to explore the impact of maternal healthy dietary habits on sleep quality during pregnancy and investigate causality and its mechanisms. Intervention studies are warranted to explore whether plant-based diets (e.g., the Mediterranean dietary pattern) might positively influence sleep quality during gestation.

## Figures and Tables

**Table 1:** Descriptive characteristics of the study participants (*n* = 150).

Variable	Mean (SD)
Age (years)	32.9 (4.6)
Pre-gestational body mass index categorization (*n* = 136)	
Normal weight (*n* %)	87 (64.0)
Overweight (*n* %)	34 (25.0)
Obese (*n* %)	11 (11.0)
16th gestational week	
Body mass index (kg/m^2^; *n* = 148)	24.9 (4.1)
Pittsburgh Sleep Quality Index global score (0–21)	6.01 (3.2)
Poor sleep quality (*n* %)	72 (48.0)
Mediterranean Food Pattern (4–35)	20.6 (5.1)
34th gestational week (*n* = 118)	
Pittsburgh Sleep Quality Index global score (0–21)	8.83 (3.76)
Poor sleep quality (*n* %)	89 (75.4)
Mediterranean Food Pattern (4–35)	21.1 (5.4)
Educational Status	*n* (%)
Non-university studies	62 (41.3)
University studies	88 (58.7)
Marital status	
Single/divorced	62 (41.3)
Married	88 (58.7)
Working status	
Not working (unemployed/homework/student/sick leave)	48 (32.0)
Part-time employment/full-time employment	102 (68.0)
Number of children	
0	90 (60.0)
1 or more	60 (40.0)
Smoking status ((yes, *n* (%))	13 (8.7)
Physical or psychological disease diagnosis ((yes, *n* (%))	61 (40.7)

Values shown as mean (SD) unless otherwise indicated. SD—standard deviation.

**Table 2:** Association between the Mediterranean Food Pattern and the Mediterranean diet components with the Pittsburgh Sleep Quality Index global score at the 16th gestational week (*n* = 150) and 34th gestational week (*n* = 118).

Food Groups	PSQI Global Score ^a^ (16th Gestational Week)	PSQI Global Score ^a^ (34th Gestational Week)
Whole-grain cereals (s/week)	−0.056	−0.158
Potatoes (s/week)	−0.012	0.099
Fruits (s/day)	−0.216 **	−0.126
Vegetables (s/day)	−0.025	−0.089
Pulses (s/week)	0.112	0.043
Fish (s/week)	0.032	−0.087
Red meat and subproducts (s/week)	0.144	0.198 *
Poultry (s/week)	0.064	0.101
Whole dairy products (s/week)	0.012	−0.094
Olive oil (s/week)	−0.162 *	−0.192 *
Nuts (s/week)	−0.096	−0.160
Sweets (s/week)	0.048	0.138
Mediterranean Food Pattern (4–35)	−0.169 *	−0.301 **

^a^ A higher score means worse sleep quality. PSQI—Pittsburgh Sleep Quality Index; s—servings. * *p* < 0.05; ** *p* < 0.01.

## Supplementary Materials

The following are available online at https://www.mdpi.com/2072-6643/12/11/3569/s1, Figure S1. Pittsburgh Sleep Quality Index global score by Mediterranean Food Pattern adherence; Table S1. Differences in the dietary habits, the Mediterranean Food Pattern and the Pittsburgh Sleep Quality Index global score of pregnant women by gestational week (16th versus 34th gestational weeks) (*n* = 117); Table S2. Association between Mediterranean Food Pattern and Mediterranean diet components with the Pittsburgh Sleep Quality Index global score at the 16th gestational week and 34th gestational week according to pre-pregnancy body mass index categorization; Table S3. Association between Mediterranean Food Pattern and Mediterranean diet components with the Pittsburgh Sleep Quality Index global score at the 16th gestation week and 34th gestational week for the control group and the intervention groups; Table S4. Differences in the Pittsburgh Sleep Quality Index global score of pregnant women at the 34th gestational week by exercise intervention (control versus intervention).
