# Supplementary material for: Influence of Dietary Habits and Mediterranean Diet Adherence on Sleep Quality during Pregnancy. The GESTAFIT Project
Source: Nutrients. 2020 Nov 20;12(11):3569. doi: 10.3390/nu12113569 (PMC7699965; doi:10.3390/nu12113569)
Supplement: Supplementary file 1 [file nutrients-12-03569-s001.pdf]

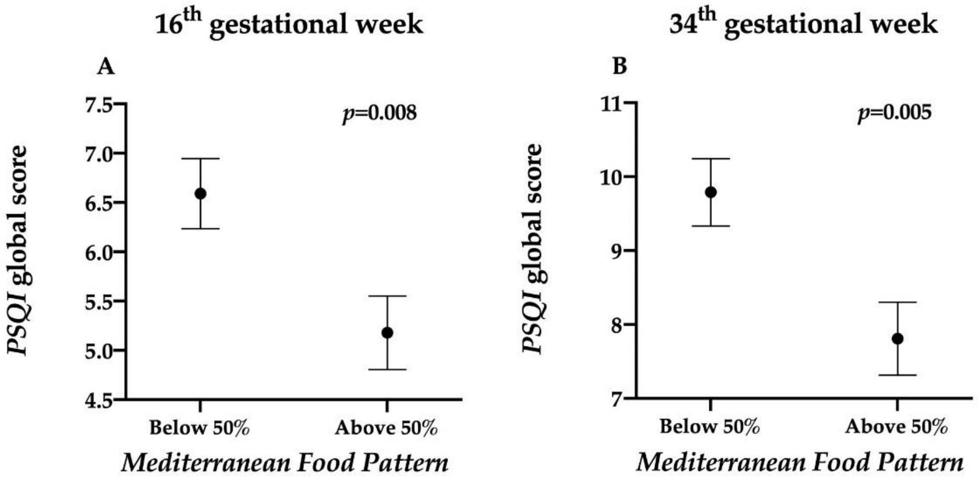

Supplementary Figure S1. Pittsburgh Sleep Quality Index global score by Mediterranean Food Pattern adherence. Dots represent mean and bars standard error. PSQI, Pittsburgh Sleep Quality Index. (A) Pittsburgh Sleep Quality Index global score by low vs. high Mediterranean Food Pattern at the 16th gestational week. (B) Pittsburgh Sleep Quality Index global score by low vs. high Mediterranean Food Pattern at the 34th gestational week.

Supplementary table S1. Differences in the dietary habits, the Mediterranean Food Pattern and the Pittsburgh Sleep Quality Index global score of pregnant women by gestational week (16th versus 34th gestational weeks) (n = 117).

	**16th week of gestation**	**34th week of gestation**	***p***
**Food frequency**			
Whole-grain cereals (s/week)	4.9 (5.3)	5.6 (6.3)	0.101
Potatoes (s/week)	2.3 (1.2)	2.3 (1.5)	1.000
Fruits (s/day)	2.1 (1.1)	2.4 (1.2)	0.010
Vegetables (s/day)	3.6 (1.7)	3.8 (1.7)	0.014
Pulses (s/week)	2.6 (1.1)	2.6 (1.1)	0.394
Fish (s/week)	4.9 (2.3)	5.3 (2.4)	0.112
Red meat and subproducts (s/week)	5.4 (3.4)	5.7 (3.8)	0.340
Poultry (s/week)	2.6 (1.2)	2.6 (1.3)	0.862
Whole dairy products (s/week)	2.9 (3.9)	4.0 (1.8)	0.044
Olive oil (s/week)	13.9 (11.6)	13.3 (7.9)	0.888
Sweets (s/week)	8.9 (6.4)	9.5 (6.9)	0.195
Nuts (s/week)	5.2 (5.3)	5.5 (5.4)	0.487
Mediterranean Food Pattern (4-35)	21.0 (5.0)	21.1 (5.4)	0.847
Pittsburgh Sleep Quality Index global score (0-21)[a] (n = 116)	6.20 (3.3)	8.76 (3.8)	<0.001

[a]A higher score means worse sleep quality. Data shown as mean (standard deviation); s—servings.

Supplementary Table S2. Association between Mediterranean Food Pattern and Mediterranean diet components with the Pittsburgh Sleep Quality Index global score at the 16th gestational week and 34th gestational week according to pre-pregnancy body mass index categorization.

	**Pre-pregnancy normal-weight**	**Pre-pregnancy overweight or obese**	**Pre-pregnancy normal-weight**	**Pre-pregnancy overweight or obese**
**Food groups**	**PSQI global scorea (16th gestational week) (*n* = 87)**	**PSQI global scorea (16th gestational week) (*n* = 49)**	**PSQI global scorea (34th gestational week) (*n* = 78)**	**PSQI global scorea (34th gestational week) (*n* = 34)**
Whole-grain cereals (s/week)	0.088	-0.202	-0.083	-0.204
Potatoes (s/week)	-0.004	-0.058	0.101	0.030
Fruits (s/day)	-0.269*	-0.083	-0.074	-0.232
Vegetables (s/day)	0.012	-0.108	-0.163	0.036
Pulses (s/week)	0.050	0.188	-0.044	0.161
Fish (s/week)	0.056	-0.016	-0.112	-0.273
Red meat and subproducts (s/week)	0.080	0.222	0.096	0.250
Poultry (s/week)	0.162	-0.028	0.075	0.119
Whole dairy products (s/week)	-0.062	0.010	-0.152	0.030
Skimmed dairy products (s/week)	0.138	-0.211	-0.020	-0.243
Olive oil (s/week)	0.006	-0.401**	-0.260*	-0.198
Nuts (s/week)	-0.009	-0.084	-0.105	-0.191
Sweets (s/week)	0.064	-0.012	0.057	0.181
Mediterranean Food Pattern (4-35)	-0.122	-0.207	-0.284*	-0.362*

[a]A higher score means worse sleep quality. PSQI—Pittsburgh Sleep Quality Index; s—servings. *$p < 0.05$; **$p < 0.01$.

Supplementary Table S3. Association between Mediterranean Food Pattern and Mediterranean diet components with the Pittsburgh Sleep Quality Index global score at the 16th gestation week and 34th gestational week for the control group and the intervention groups.

	Control group	Control group	Intervention group
**Food groups**	**PSQI global scorea (16th gestational week) (*n* = 78)**	**PSQI global scorea (34th gestational week) (*n* = 52)**	**PSQI global scorea (34th gestational week) (*n* = 66)**
Whole-grain cereals (s/week)	-0.025	-0.107	-0.189
Potatoes (s/week)	-0.073	0.234	-0.028
Fruits (s/day)	-0.292**	-0.214	-0.035
Vegetables (s/day)	-0.025	-0.157	-0.027
Pulses (s/week)	0.096	-0.027	0.089
Fish (s/week)	-0.036	-0.175	-0.036
Red meat and subproducts (s/week)	0.142	0.169	0.236
Poultry (s/week)	0.096	0.139	0.059
Whole dairy products (s/week)	-0.159	-0.099	-0.005
Skimmed dairy products (s/week)	0.078	-0.035	-0.134
Olive oil (s/week)	-0.281*	-0.367**	-0.006
Nuts (s/week)	-0.208	-0.225	-0.085
Sweets (s/week)	-0.009	0.165	0.118
Mediterranean Food Pattern (4-35)	-0.240*	-0.451**	-0.150

[a]A higher score means worse sleep quality. PSQI—Pittsburgh Sleep Quality Index; s—servings. *p<0.05; **p<0.01.

Supplementary Table S4. Differences in the Pittsburgh Sleep Quality Index global score of pregnant women at the 34th gestational week by exercise intervention (control versus intervention).

	**Control group (*n* = 52)**	**Intervention group (*n* = 66)**	***p***
PSQI global score (0-21)[a]	8.96 (3.8)	8.73 (3.7)	0.726

[a]A higher score means worse sleep quality. PSQI—Pittsburgh Sleep Quality Index.
